# Diagnostics for optimised dengue surveillance: a qualitative focus group study to investigate user experience and requirements in Thailand

**DOI:** 10.1136/bmjopen-2024-085946

**Published:** 2024-11-20

**Authors:** Paul Arkell, Sanhapon Ketklao, Adisak Songjaeng, Dumrong Mairiang, Jesus Rodriguez-Manzano, Pantelis Georgiou, Alison Holmes, Raheelah Ahmad, Prida Malasit, Panisadee Avirutnan, Saranath Lawpoolsri

**Affiliations:** 1 Centre for Antimicrobial Optimisation, Imperial College London, London, UK; 2 Siriraj Center of Research Excellence in Dengue and Emerging Pathogens (SiCORE-Dengue), Faculty of Medicine Siriraj Hospital, Mahidol University, Bangkok, Thailand; 3 Division of Dengue Hemorrhagic Fever Research, Faculty of Medicine Siriraj Hospital, Mahidol University, Bangkok, Thailand; 4 Molecular Biology of Dengue and Flaviviruses Research Team, Medical Molecular Biotechnology Research Group, National Center for Genetic Engineering and Biotechnology (BIOTEC), National Science and Technology Development Agency (NSTDA), Bangkok, Thailand; 5 Department of Electrical and Electronic Engineering, Imperial College London, London, UK; 6 Department of Health Services Research & Management, City St George's, University of London, London, UK; 7 Department of Tropical Hygiene, Faculty of Tropical Medicine, Mahidol University, Bangkok, Thailand

**Keywords:** Diagnostic microbiology, Epidemiology, Molecular diagnostics, Public health, Tropical medicine

## Abstract

**ABSTRACT:**

**Objectives:**

Effective, real-time surveillance of dengue may provide early warning of outbreaks and support targeted disease-control intervention but requires widespread accurate diagnosis and timely case reporting. Research directing innovation in diagnostics for dengue surveillance is lacking. This study aimed to describe experience and requirements of relevant prospective users.

**Design:**

A qualitative, focus group study was conducted.

**Participants:**

Data were collected from 19 users of diagnostic technology who work across the Thai dengue surveillance system.

**Data collection and analysis:**

Contextual knowledge, experience and needs were explored in focus groups. Discussions were translated, transcribed, analysed thematically and mapped to Consolidated Framework for Implementation Research domains.

**Results:**

Participants expressed a need for rapid, accurate, serotype-specific tests which can be operated easily by non-expert users without laboratory equipment. They supported integration of diagnostics with surveillance systems and felt this would increase the quantity and speed of case reporting as well as provide healthcare professionals with up-to-date information about the number of cases locally, thereby aiding interpretation of test results. Concerns included those relating to data security and the cost of tests.

**Conclusions:**

Engagement to understand prospective user experience and requirements can improve relevance and uptake of new technology, leading to system efficiencies. The present study highlights specific needs for accurate, serotype-specific, remote-connected diagnostics which are integrated with surveillance systems and support dengue case reporting at the point-of-care.

**STRENGTHS AND LIMITATIONS OF THIS STUDY**

- Specific investigation into user requirements for diagnostics which support dengue surveillance.
- Included technology users in Thailand with wide ranging professional experience including operation of tests and downstream analysis/usage of data.
- Thematic analysis with mapping to Consolidated Framework for Implementation Research domains.
- Only included participants working within one national surveillance system and excluded patients and the general public who also play pivotal roles as users.

## Introduction

 Dengue is a mosquito-borne neglected tropical disease which affects 100–400 million individuals annually and is a significant cause of morbidity and mortality among adults and children. It is caused by four dengue virus serotypes (DENV1–4) which co-circulate in many regions.^1^ Dengue causes a diverse clinical syndrome ranging from asymptomatic or mild, self-limiting illness to dengue haemorrhagic fever, dengue shock and death.^2 3^ ‘Secondary dengue infection’, which occurs when an individual is infected for a second (or subsequent) time by a different serotype to their earlier ‘primary infection’, is most likely to result in severe disease.^4^

A diagnosis can be suspected based on clinical features and routinely available laboratory data but should be confirmed using a diagnostic test.^3^ Reverse-transcriptase polymerase chain reaction (RT-PCR) assays detect dengue RNA. They have high sensitivity and specificity, are considered the modern reference standard diagnostic test and may be used to serotype infections.^5^ However, RT-PCR requires significant laboratory infrastructure and a skilled workforce, resulting in its limited use in rural and remote locations.^6^ Serological techniques (including enzyme-linked immunosorbent assays (ELISAs)) can be used to detect host immunoglobulins M and G and virus proteins (non-structural protein 1, NS1). Similar to RT-PCR, laboratory-based serological testing has been challenging to deploy. Therefore, rapid diagnostic tests (RDTs), which also detect immunoglobulin M, immunoglobulin G and/or NS1, are more commonly used in rural and remote locations. These are low cost and simple to use but have varying sensitivity compared with RT-PCR (40% to >90%) and ELISA, which depends on time since onset of symptoms. Current RDTs cannot determine the infecting serotype.^7^

Outbreaks of dengue are typically seasonal with the number of cases and proportion causing severe disease being highly variable between years. Shifts in the predominant circulating serotype may lead to more severe outbreaks.^8^ In ‘passive surveillance’, cases are identified via the routine assessment of unwell patients at healthcare facilities and are notified to a central surveillance authority. This relies on availability and utilisation of accurate diagnostic tests and effective, timely communication of results alongside clinically derived metadata. Passive surveillance may be augmented at ‘sentinel sites’, with samples undergoing additional serotype-specific testing.^9 10^ Effective implementation of such systems with real-time data transfer may provide early outbreak warning.^9^,^12^ However, common weaknesses include poor access to diagnostic testing and delayed or incomplete reporting.^9 13^ In Thailand, there is mandatory reporting of clinical or RDT-confirmed cases to regional surveillance authorities by healthcare facilities.

Several advances in diagnostic technology represent opportunity to enhanced dengue surveillance.^14^ Novel molecular techniques such as reverse-transcriptase loop-mediated isothermal amplification may lead to high-sensitivity portable diagnostic devices for detecting and serotyping infections.^15 16^ Mobile phone and global positioning system technologies may be integrated to automate case notification.^12 17 18^

In the context of dengue surveillance, ‘users’ of technology include those involved in the operation and interpretation of diagnostic devices and/or the use of data generated to make decisions about management of individual patients and population level surveillance or disease control.^19^ The professional occupation of individuals undertaking these activities varies between country and healthcare setting, but may include public health practitioners, surveillance officials, doctors, nurses and laboratory scientists. Patients and the general public also play pivotal roles as users. Research into user requirements for diagnostics to enhance dengue surveillance is lacking. Previous studies evaluating the implementation of existing RDTs for other pathogens have identified some potential barriers from the perspective of users. These include unreliable supply chains, user training requirements, practical limitations in operating devices, difficulties interpreting and recording results, distrust of results, and a lack of impact on clinical decision-making.^20^,^24^ Beyond infectious disease diagnosis and surveillance contexts, there is frequent non-adoption of health technology, including in rural and remote settings.^19 25 26^ It is crucial that technology is developed and evaluated in collaboration with intended users. Engagement throughout the design process likely results in optimised solutions and maximised chances of technology adoption.^27^ The Consolidated Framework for Implementation Research (CFIR) provides a set of domains which can be used to systematically assess barriers and facilitators to implementing health intervention. These include the intervention itself and how it may be adapted, the setting, the processes and individuals involved.^28^

This study engaged users of diagnostic technology working across the Thai dengue surveillance system. It explored their contextual knowledge, experience and needs, with the aim of determining requirements for new devices and their implementation in systems of dengue surveillance.

## Methods

### Setting

This qualitative study was conducted during July 2022 at four institutions in Thailand: The Division of Vector Borne Diseases, Department of Disease Control (CDC) at the Ministry of Public Health is the national authority responsible for surveillance of dengue and strategies for dengue control. The Hospital for Tropical Diseases (HTD) is a tertiary care hospital specialised in tropical diseases including dengue. Khon Kaen Hospital (KKH) is a public hospital which provides inpatient and outpatient care for rural patients. The Dengue Haemorrhagic Fever Research Unit at Mahidol University (DHFRU), Bangkok, is an academic centre with a multidisciplinary dengue research portfolio.

### Participants

A purposive sample was taken to ensure inclusion of participants with a range of experience across dengue surveillance in Thailand. This included public health practitioners, surveillance officials, doctors, nurses, laboratory scientists and dengue researchers. One focus group containing at least two of these professional groups was constructed at each of the above institutions. Participants were identified via their professional relationships with research team members and were approached during their usual working day.

### Data collection

Data were collected during four focus group discussions, each including between four and seven participants. These were facilitated by two researchers and were conducted either in English or Thai language, depending on participant preference. Discussion was facilitated using a topic guide, developed in advance based on literature review and expert’s opinion regarding knowledge and innovations in dengue diagnosis and surveillance (box 1). This was reviewed and revised iteratively during and between sessions to ensure that emerging themes could be identified, explored further, and triangulated within and between groups of participants. Focus groups were audio-recorded, and written notes were taken. Recordings were transcribed, and Thai was translated to English language.

Box 1 **Focus group topic guide**

- Contextual understanding and needs assessment.
  - How is dengue surveillance done, at your workplace (and more broadly)?
  - Where do patients present to with symptoms of dengue and how do they get diagnosed?
  - If tests are not always done, why do you think this is?
  - Where/how should cases of dengue get reported, to surveillance?
  - If positive results are not always reported, why do you think this is?
  - How are surveillance data used?
- Requirements for new diagnostic devices: the assay.
  - Where does diagnostic testing usually occur, and what laboratory equipment is available there (if any)?
  - Who typically operates diagnostic devices, and what sample preparation/analysis skills do they have (if any)?
  - What do you think would be the preferred sample type and sample volume that would go into any new diagnostic device?
  - What do you think would be the preferred (and maximum) time from sample to result (ie, test duration) of any new diagnostic device?
  - What do you think the preferred (and minimum) sensitivity and specificity of any new diagnostic device?
  - Is knowing the dengue serotype important?
  - Is knowing the quantity of dengue (level of ‘viraemia’) in a patient’s sample important?
- Requirements for new diagnostic devices: remote connectivity and reporting.
  - How are results from diagnostic tests generally reported, and where are they stored?
  - If a new diagnostic device could be remote-connected, where should results be reported to?
  - Which information about cases would be most useful to report alongside test results to enhance dengue surveillance?
  - Would it be useful if a new diagnostic device could receive and display real-time information about local dengue incidence to the user (as well as transmitting data for case reporting)?

### Data analysis

A thematic analysis was undertaken.^29^ Transcripts from each focus group were annotated and analysed by two researchers who assigned codes independently and then discussed and aggregated them into themes. A deductive approach was used, with themes mapped to CFIR domains.^28 30^ ‘Current practices and challenges’ and ‘requirements for new diagnostics in surveillance’ were overarching themes agreed a priori, as they were central to the aim of the study.

### Ethical considerations

Potential participants received verbal and written information about the proposed study purpose and its procedures. All participants provided written informed consent. This study received ethical approval from Mahidol University Faculty of Tropical Medicine Research Ethics Committee (MUTM-2022-031-01).

### Patient and public involvement

This current phase of research and development did not include patients or public representatives.

## Results

Nineteen individuals participated, 12 of whom were female. These worked at HTD (6), DHRFU (5), KKH (4) and CDC (4). They included nurses (5), doctors (4), dengue researchers (4), laboratory scientists (2), public health practitioners (2) and surveillance officials (2).

**Figure 1:**
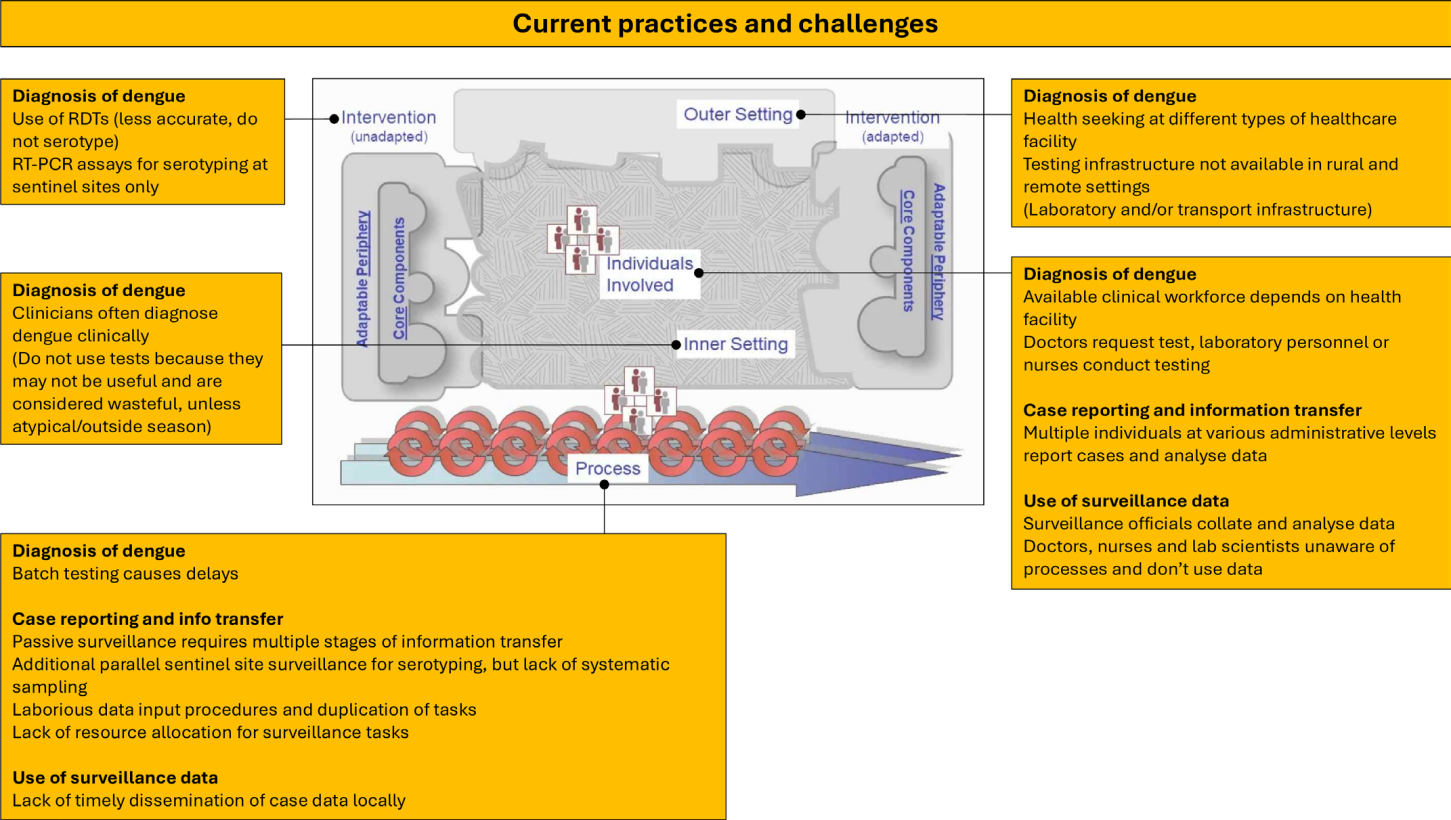
Identified themes within ‘current practices and challenges relating to dengue diagnosis and surveillance’ mapped to the Consolidated Framework for Implementation Research (CFIR) domains. Inner figure reproduced with permission from the original open access publication, available at: https://cfirguide.org/cfirdiagram/.

**Figure 2:**
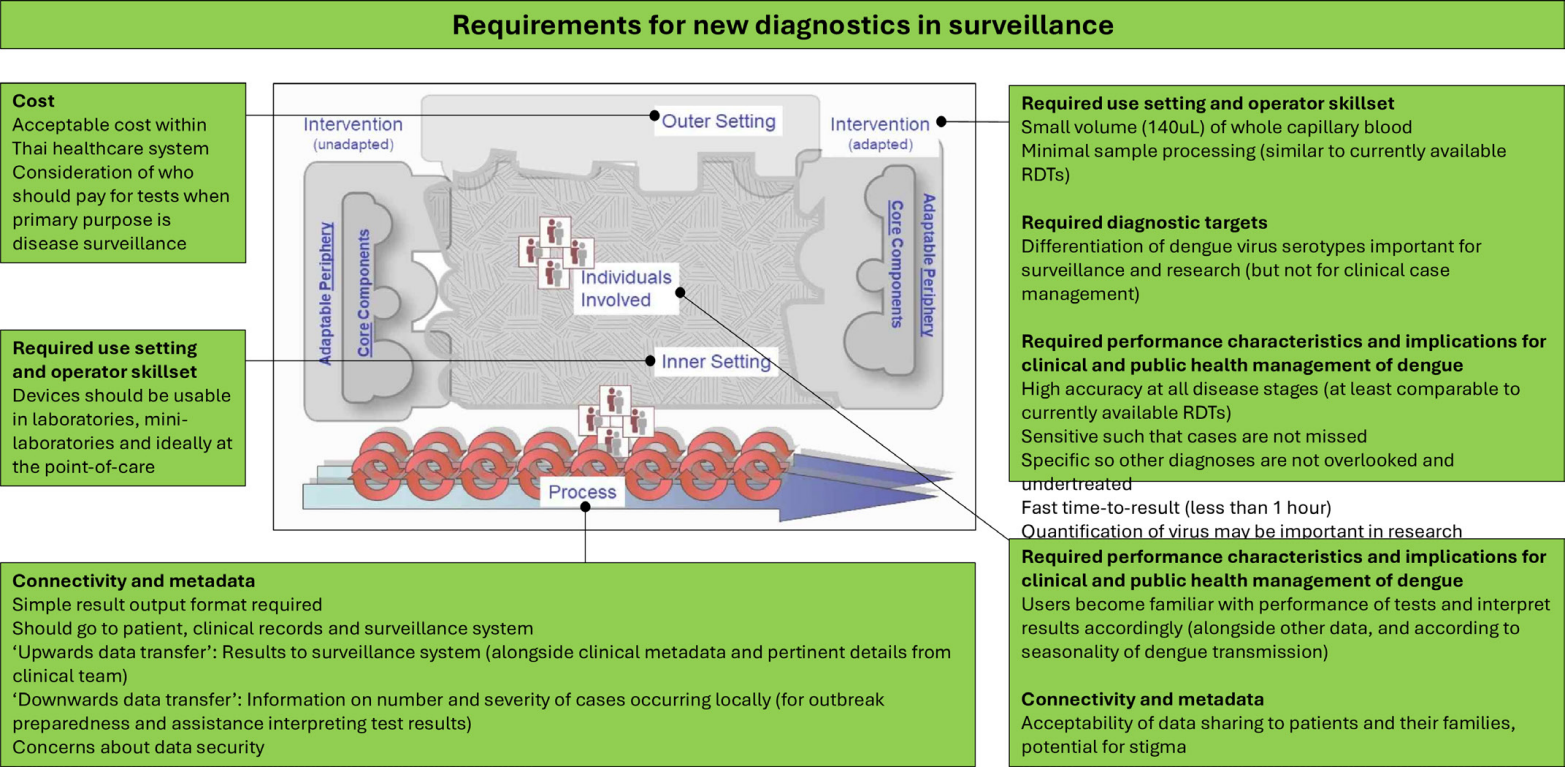
Identified themes within ‘requirements for new diagnostics’ mapped to the Consolidated Framework for Implementation Research (CFIR) domains. Inner figure reproduced with permission from the original open access publication, available at: https://cfirguide.org/cfirdiagram/.

Identified themes mapped to the CFIR (figure 1) demonstrate barriers across all parts of the system including the poor fit between current technologies and adopting context. Features likely to address these barriers (figure 2) are also identified, providing viable design and implementation approaches. These are further described and supported by selected quotations from participants below.

### Current practices and challenges

#### Diagnosis of dengue

Participants described how individuals with dengue may seek healthcare at different types of healthcare facility, including primary health centres, district hospitals, regional hospitals, referral hospitals, pharmacies or private clinics, with each type having different clinical workforce and diagnostic test availability. There is a lack of diagnostic testing in many rural and remote settings.

*It depends on the level of [healthcare facility], if located in a very remote area, they cannot do a blood test.*

- Participant 6, Laboratory Scientist. Focus group 2.

Senior doctors described frequently diagnosing dengue based on clinical features, and many said they often did not use a diagnostic test.

*I think the senior doctors like me are very used to following the clinical, but I think the new generation of doctors are more likely to use the [RDT].*

- Participant 13, Doctor (Paediatrics). Focus group 3.

Cited reasons for not testing included a high degree of confidence in clinical diagnoses, potentially inaccurate tests and resource wasting. Some reported only using tests in atypical cases or outside dengue season.

When tests are used, RDTs are operated at laboratories or ‘mini laboratories’ (non-clinical areas attached to smaller healthcare facilities) by a laboratory scientist or sometimes at the point-of-care by a nurse. RT-PCR is rarely used because samples (or patients themselves) must be transported to specialist laboratories and results may be delayed.

#### Case reporting and information transfer

**Figure 3:**
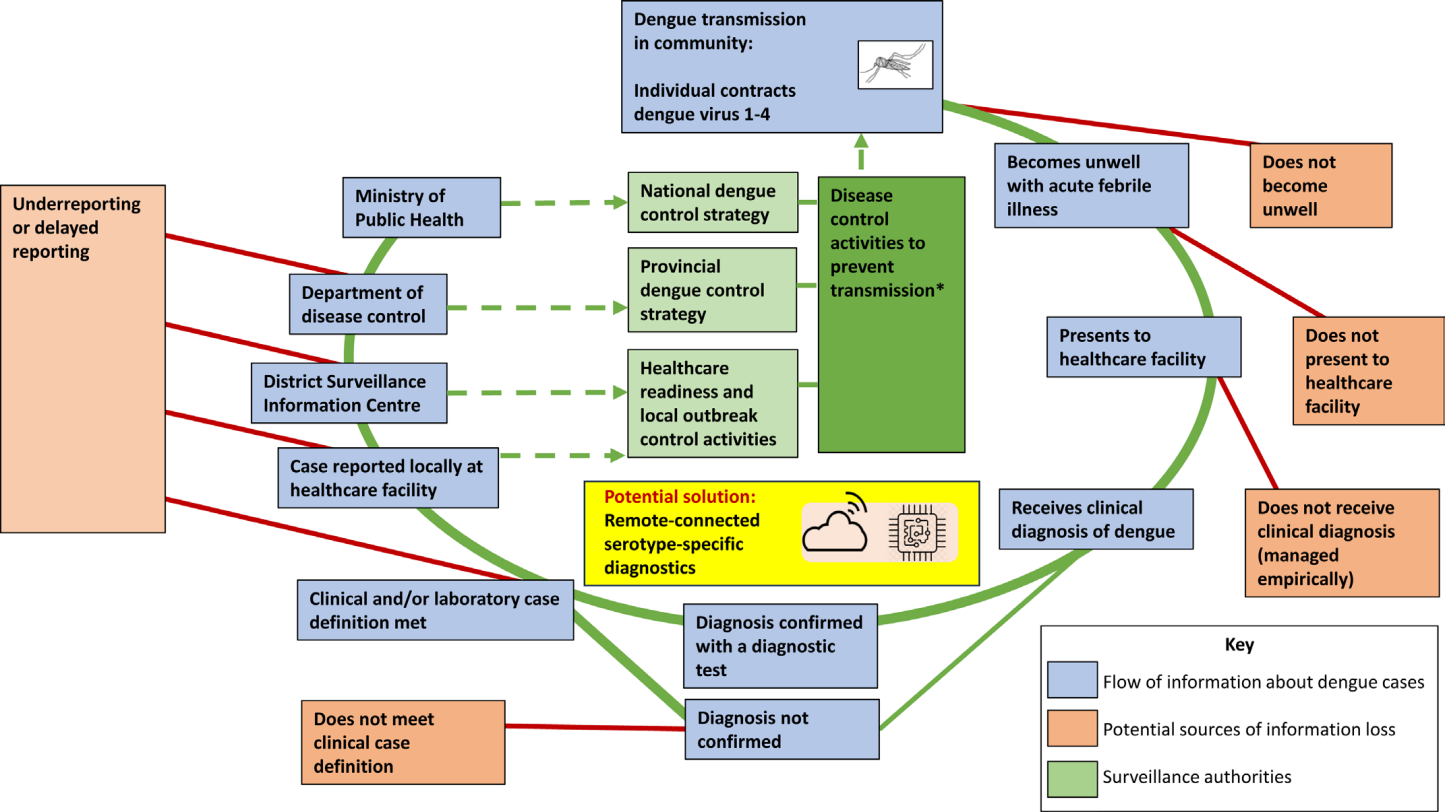
Schematic diagram showing current, multi-level transfer of information in a passive dengue surveillance system. Information is predominantly transferred ‘upwards’, with limited ‘downwards data transfer’ to communities and users. *Current disease control activities comprise environmental management and insecticide use. Future activities may also include deployment of vaccines and Wolbachia-infected mosquitos.

Participants described a system of passive disease surveillance requiring multiple stages of information transfer. Typically, diagnosed cases of dengue are communicated to an individual with responsibility for disease reporting at a health facility. Information is then transferred sequentially to local, regional and national levels of the surveillance system (figure 3).^31^ This can be written on paper forms which are transferred manually between individuals and departments.

This information transfer could be incomplete or delayed, potentially by up to 4 weeks, due to laborious data input procedures, frequent duplication of tasks and lack of time and resource allocation for these activities.

*Oh I’m really sad…to tell you, not only do we have an underdiagnosis situation, but we have an underreporting situation also.*

- Participant 17, Senior Surveillance Official. Focus group 4.

*One of the reasons they don’t report is they have to sit down and key in the result.*

- Participant 18, Surveillance Official. Focus group 4.

Some participants also described a parallel sentinel site surveillance system, with samples undergoing serotype-specific testing at a central location. However, only low numbers of cases are included, these are not recruited systematically and batch-testing results in availability of serotype data being delayed.

#### Use of surveillance data

When participants were asked about the benefits of case reporting, responses varied according to professional occupation. Doctors, nurses and laboratory scientists did not identify benefits from this activity and were unaware of downstream processes . They rarely received epidemiological information or warning about outbreaks as a result participation in surveillance.

*No one tells us, we just know when a large number of patients is coming!*

- Participant 13, Doctor (Paediatrics). Focus group 3.

Public health practitioners and surveillance officials explained how national and regional data are collated into reports but agreed that information could be disseminated more rapidly and used more efficiently locally.

### Requirements for new diagnostics in surveillance

#### Use setting and operator skillset

Participants stated that new devices for the diagnosis of dengue should be usable in a wide range of settings, including at the point-of-care (inpatient and outpatient) and in laboratories and ‘mini laboratories’. There was a preference for analysing a small volume (up to four drops, ~140 uL) of capillary blood, obtainable by finger-prick and transferred directly into the device.

*If we use it in outpatients where there are many patients, obtaining blood from the fingertip would be suitable.*

- Participant 14, Nurse (Inpatient). Focus group 3.

There was a strong desire for minimal sample processing prior to analysis (ie, centrifugation, pipetting, mixing or addition of reagents). This was frequently explained by reference to currently available RDTs, which are simple to use.

*Nurses are not using pipette. If that’s needed, it needs to be in the lab.*

- Participant 7, Nurse Assistant (Outpatients). Focus group 2.

*We have to try to mimic the [RDTs].*

- Participant 3, Dengue Researcher. Focus group 1.

#### Diagnostic targets

Many participants stated that new diagnostic devices should have the ability to serotype infections. Public health practitioners, surveillance officials and several dengue researchers had particularly strong desires for this, noting that it has not been achieved by currently available RDTs.

*If we can get the serotype in real-time of course it will make our control measures more effective.*

- Participant 19, Surveillance Official. Focus group 4.

Doctors and nurses could also understand this potential surveillance benefit but stated that serotypes are of little consequence for individual patient management.

#### Assay performance characteristics and implications for clinical and public health management of dengue

Most participants cited ‘accuracy’ as an important characteristic. They recognised that existing dengue tests sometimes had low sensitivity, which could affect patient management as well as surveillance. Low sensitivity tests which give falsely negative results may lead missed diagnoses of dengue, with further testing and treatments for other causes (eg, bacterial infections) being initiated or continued unnecessarily.

*If the doctors see that the test is negative, [they] might diagnose something else and treat something else, like bacterial infection… [this] might harm the patient.*

- Participant 10, Doctor (Internal Medicine). Focus group 4.

They suggested that new devices should have at least the same sensitivity as currently available RDTs.

Participants also recognised that non-specific tests could lead to alternative diagnoses being missed and discontinuation of important treatments (eg, antibiotics).

*If it has false positive it may lead to mistreatment of other diseases.*

- Participant 16, Doctor (Internal Medicine). Focus group 3.

*This means it’s not dengue but something else. Yes definitely, this delays the treatment. Yes it’s going to be a problem.*

- Participant 10, Doctor (Internal Medicine). Focus group 4.

They caveated this by suggesting that users would become familiar with the performance of any new test and would interpret results accordingly. They also described how clinical and epidemiological context are considered, when interpreting dengue test results.

*We use it along with [routine laboratory data]. If [the test] is negative, but the case is likely to be dengue, we still have [routine laboratory data] to follow-up the patient.*

- Participant 16, Doctor (Internal Medicine). Focus group 3.

*If the local prevalence of the infection is high, then the test-negative will not ensure that the patient has no dengue infection. But if the patient is in a without dengue area, we will have high confidence that this patient does not have dengue infection. It will depend on the prevalence at the time and in the local area.*

- Participant 16, Doctor (Internal Medicine). Focus group 3.

Many participants also cited ‘fast result’ as an important characteristic. This was particularly important for nurses and laboratory scientists who are frequent operators of RDTs. They suggested target sample-to-result time should be below 1 hour (and ideally below 15–20 min).

The ‘ability to quantify virus’ was not considered an important characteristic, either for clinical or surveillance purposes. However, some participants acknowledged potential utility in clinical research, for example, in trials of antiviral mediations.

#### Connectivity and metadata

Participants recommended that diagnostic devices should have a simple way of displaying results to users with low chance of misinterpretation. They also stated that results should be recorded permanently on a patient’s record. This could be achieved by integrating devices with electronic patient records and/or laboratory information systems, or by allowing results to be printed.

There was agreement among all participants that integrating diagnostic devices with surveillance systems could be helpful and that receiving serotype data would support surveillance efforts. Many suggested that it would reduce requirements for informal communication, paper records, data input and duplication of work at several levels of the surveillance system, hence improving case reporting. Public health practitioners and surveillance officials detailed which metadata should be reported routinely alongside the test results (box 2). They also felt that optional reporting of pertinent clinical details could be useful (eg, details of particularly severe or atypical cases which may warrant further investigation).

Box 2 **Basic metadata requirements for automated case reporting within the Thai surveillance system.**

**Upwards data transfer (device to surveillance system)**

- Test-related data
  - Date of test (date).
  - Geo-location of test (lat, long).
  - Dengue test result (positive/negative).
  - Serotype result (DENV1/DENV2/DENV3/DENV4).
- Identifiers
  - Name (free text).
  - National ID (number).
  - Home address (free text).
  - Patient’s (or parent/guardian’s) telephone number (number).
- Clinical details
  - Duration of symptoms in days (number).
  - Severity of case at time of testing if dengue suspected clinically (non-severe/dengue with warning signs/severe dengue/patient died).
  - Alternative clinical diagnos(es), if applicable (free text).
  - Additional information for communication to surveillance authority. For example, details of particularly severe or atypical cases or those where multiple family members are unwell, which may warrant further investigation (free text).

**Downwards data transfer (surveillance system to device)**

- All test-related data (see A, above) from other devices.*

* These data could be output to the clinical user as individual cases (for exampleeg, displayed on a map), or after aggregation and/or analysis in the form of an epidemiological report.

As well as performing automated case notification (‘upwards data transfer’), participants suggested that a new diagnostic device could also receive and display epidemiological data to the user (‘downwards data transfer’). They expressed their desires for up-to-date information about the numbers and severity of dengue cases in their area and agreed that devices which provide early warning of dengue outbreaks would be useful.

*If we know the information about the outbreak of dengue cases in the surrounding area, we will be more aware of the possibility of more severe cases coming to the hospital*.

- Participant 13, Doctor (Paediatrics). Focus group 3.

Some explained how this knowledge could be used to assist in the interpretation of the dengue test itself.

*When patients present with fever during the outbreak season the clinician usually ask where they come from. If we know that they come from an outbreak area, it increases the possibility that the case may be dengue*.

- Participant 13, Doctor (Paediatrics). Focus group 3.

However, some participants had concerns relating to data security, particularly if devices could receive, store or display potentially sensitive information about other cases in the region (eg, their location).

*Someone can think about stigmatisation. OK so this family has dengue and someone can think that they are spreading dengue to the village, or something like that.*”

- Participant 10, Doctor (Internal Medicine). Focus group 2.

#### Cost

Participants emphasised the importance of cost when considering the potential introduction of new diagnostic devices in Thailand. Usually, diagnostic testing is paid for by government insurance coverage, private insurance or personal funds. Many participants considered a conceptual difference between testing which is undertaken for individual patient benefit (ie, for diagnostic purposes) and that which is undertaken for potential collective population benefit (ie, for surveillance) and felt that using personal funds to pay for the latter would be unfair.

## Discussion

Participants in this study identified the need and potential value of new tests for dengue which are accurate, rapid and low cost and can be operated easily by non-expert users outside laboratory settings, including in remote and rural areas. They supported integration of diagnostic devices with surveillance systems to increase quantity and speed of case notification. These requirements align with the WHO Special Program for Research and Training in Tropical Diseases ‘ASSURED’ criteria for diagnostics and subsequent publications supporting real-time connectivity (‘REASSURED’ characteristics).^32^,^34^ Tests which can serotype may be important for surveillance but are less likely to benefit individual patients. ‘Upwards data transfer’ (such that cases are easily or automatically notified by users via devices to the surveillance authority), as well as ‘downwards data transfer’ (such that local case data and outbreak information are returned to users) were considered useful potential functions. The latter would assist in interpretation of individual test results and could give early warning of outbreaks. It is likely that individual devices would individually connect with a cloud where data is stored and analysed and that this would be hosted by the local surveillance authority. Cautions relating to this overall approach included data security and the potential cost when compared with currently available diagnostic tests. Additionally, remote-connected devices which transmit and receive data may become complicated to use, potentially affecting uptake. Participants in this survey had a strong preference for diagnostics which are simple to use. Therefore, prospective technology users should be engaged and involved in design, and care must be taken to maintain simplicity and usability of devices for their primary purpose of dengue diagnosis.

Previous studies have explored healthcare workers’ and community members’ perceptions of new diagnostic devices for tropical infections, particularly those intended to be used at the point-of-care. Diggle *et al* investigated malaria RDTs in Northern Kenya and found significant knowledge gaps, misconceptions and evidence of low uptake. Reasons included perceptions that testing was unnecessary, distrust of results, fear that devices might also test for other, potentially stigmatised conditions and cost. However, RDTs were noted for their ease of use and portability.^21^ Rasti *et al* investigated Southwestern Ugandan healthcare workers who described point-of-care tests improving diagnosis and clinical decision-making in under-resourced areas. However, they also reported experiencing inaccurate results and a need to interpret and corroborate results with other clinical information.^23^ Boadu *et al* identified influencers of malaria RDT implementation among primary healthcare providers in central Ghana. These included healthcare delivery constraints, provider perceptions and social dynamics of care delivery.^20^ A scoping review of the use of mobile phones in the prevention and control of arboviral infections identified six studies where mobile phone technology formed part of a diagnostic workflow and 25 studies where mobile telephones were used in various surveillance activities.^17^ Cited benefits were a ‘reduction in error of transcribed data’, ‘rapid data transfer’ and ‘good completeness in terms of more dengue case reporting’, which are highly relatable to the present study’s findings.^17^ Another recent article has reviewed various digital health interventions which have been used in dengue surveillance.^35^

This study is the first to specifically investigate user requirements for diagnostic devices that would optimise dengue surveillance. It collected data from a wide range of diagnostic technology users, including those who make decisions to test, those with hands-on experience of operating tests and those who are involved in downstream analysis and usage of data. Broad inclusion appears to have been important because user requirements sometimes varied between occupational groups. Innovation in technology should account for this and may need to balance priorities of different users.

Limitations of this study include its restriction to 19 participants in one country, which could mean that findings are geographically specific and are not fully representative nor transferrable to other settings. However, many of the practices and challenges described appear similar to those experienced in other Southeast Asian nations^9^ and more widely.^13^ Additionally, it did not include patients or members of the general public, who are important users of diagnostic technology. In Thailand, there has been rapid increase in the use of mobile phone technology, including for storage and sharing of personal health records.^36 37^ Results from the present study highlight further need to engage this group, particularly around the importance of data security. Furthermore, this study focused on dengue, but there is likely to be significant overlap in the experiences and requirements of individuals who undertake surveillance of other arboviruses and other infectious disease more generally. Surveillance requirements for devices which may simultaneously detect multiple relevant pathogens should also be investigated, as diagnostic technology advances.

Dengue is a major public health concern across tropical regions. Accurate, serotype-specific, remote-connected diagnostic devices which can be used in a diverse range of settings would enhance surveillance and could support real-time outbreak risk assessment and warning. These should be developed in collaboration with a range of prospective technology users.

## Data Availability

Data are available upon reasonable request.
