# Supplementary material for: Diagnostics for optimised dengue surveillance: a qualitative focus group study to investigate user experience and requirements in Thailand
Source: BMJ Open. 2024 Nov 20;14(11):e085946. doi: 10.1136/bmjopen-2024-085946 (PMC11580300; doi:10.1136/bmjopen-2024-085946)
Supplement: Supplementary file 1 [file bmjopen-2024-085946.reviewer_comments.pdf]

PEER REVIEW HISTORY

BMJ Open publishes all reviews undertaken for accepted manuscripts. Reviewers are asked to complete a checklist review form and are provided with free text boxes to elaborate on their assessment. These free text comments are reproduced below.

ARTICLE DETAILS

Title (Provisional)

Diagnostics for optimised dengue surveillance: A qualitative focus group study to investigate user experience and requirements in Thailand

Authors

Arkell, Paul; Ketklao, Sanhapon; Songjaeng, Adisak; Mairiang, Dumrong; Rodriguez-Manzano, Jesus; Georgiou, Pantelis; Holmes, Alison; Ahmad, Raheelah; Malasit, Prida; Avirutnan, Panisadee; Lawpoolsri, Saranath

---

VERSION 1 - REVIEW

**Reviewer**	**1**
**Name**	**Sulistyawati, Sulistyawati**
**Affiliation**	**Universitas Ahmad Dahlan, Public Health**
**Date**	**30-Mar-2024**
**COI**	**None**

Dear Editors and Reviewers,

This article has good results and should be widely disseminated as learning. However, several things need to be added and completed so that it can be read more comprehensively.

****The reviewer provided a marked copy with additional comments. Please contact the publisher for full details.**

**Reviewer**	**2**
**Name**	**Rojas, Alejandra**
**Affiliation**	**Instituto de Investigaciones en Ciencias de la Salud, Universidad Nacional de Asunción, Production**
**Date**	**27-Apr-2024**
**COI**	**I declare that I have no conflict of interests**

Title: Diagnostics for optimised dengue surveillance:Investigating user experience and requirements

Argel and collaborators present a study to identify requirements for new diagnostics in order to enhance dengue surveillance. This work describes the current practices and challenges related to dengue diagnosis. Furthermore, the study highlights specific needs/requirements for new dengue diagnostics.

Some minor recommendations for clarifications and adjustments in the formatting are suggested.

Keywords

Please check the MeSH terms

Introduction

Line 73. WHO classification might be cited instead.

Methods

line 141. Add the total of participants (mentioned in the abstract): "Data were collected from 19 users …"

I think it would have been interesting/useful adding some information regarding the type of tests used (RT-PCR, RDTs, assays for antibody detection like ELISAs). Considering the results, most of the participants refer to RDTs.

Also, information regarding the types of tests to be used according the number of days after onset of symptoms, and the interpretation of the results according to that (i.e. False negatives)

Regarding case reporting and information transfer (Line 195): Is an epidemiological form/questionnaire used to collect data? Is this done on paper or online? How does communication with the surveillance system occur? how are the communication steps carried out?

Results

Line 266: It is suggested to use the phrase "low sensitivity tests"

Discussion

It is noteworthy that some physicians do not request laboratory tests because other arboviruses (Zika, chikungunya, etc.) have been reported in Thailand, as well as other pathogens that can cause clinical pictures similar to dengue, highlighting the importance of differential diagnosis. Perhaps this topic could be elaborated in the discussion.

With respect to the data transfer, it could be added that certain technologies have been used for dengue surveillance. i.e. "Digital Health Interventions in Dengue Surveillance to

Detect and Predict Outbreak: A Scoping Review” (DOI: 10.2174/0118749445283264240116070726).

Figures

- Figures 1 and 2 show very interesting/relevant data. For clarity, the layouts of these images could be improved, (scheme/diagram and colors) Also, not all components of the background figure are visible and clear.

- Figure 3. Please add the meaning of AFI abbreviation (in the figure caption/footnote)

The “remote-connected serotype-specific diagnostics” box should be connected to some process/box in the figure?

You might want to add to this figure that currently the information does not go back (downwards) to the users (physicians, nurses, researchers).

What type of activities is done for disease control/prevent transmission?

Limitations

It could be added that the findings are limited to the 19 participants interviewed and so future research examining this topic in in a larger number of participants (representative of the different occupational groups) may have a broader scope.

Formatting

For the final version, could references along the manuscript be included in abbreviated form?

i.e: [20], [21], [22], [23], [24] could be cited as 20-24

Some comments were included in the manuscript.

Item 15. Is the standard of written English acceptable for publication? I cannot fully evaluate this topic because I am not a native speaker.

****The reviewer provided a marked copy with additional comments. Please contact the publisher for full details.**

VERSION 1 - AUTHOR RESPONSE

Reviewer: 1
Dr. Sulistyawati Sulistyawati, Universitas Ahmad Dahlan
Comments to the Author:
Dear Editors and Reviewers,
This article has good results and should be widely disseminated as learning. However, several things need to be added and completed so that it can be read more comprehensively.

Thank you for your constructive feedback. All comments on the PDF have been addressed

Reviewer: 2
Dr. Alejandra Rojas, Instituto de Investigaciones en Ciencias de la Salud, Universidad Nacional de Asunción
Comments to the Author:
Title: Diagnostics for optimised dengue surveillance:Investigating user experience and requirements

Argel and collaborators present a study to identify requirements for new diagnostics in order to enhance dengue surveillance. This work describes the current practices and challenges related to dengue diagnosis. Furthermore, the study highlights specific needs/requirements for new dengue diagnostics.

Some minor recommendations for clarifications and adjustments in the formatting are suggested.

Keywords
Please check the MeSH terms

Thank you. Keywords have been updated to MeSH terms where possible.

Introduction
Line 73. WHO classification might be cited instead.

This citation has been added

Methods
line 141. Add the total of participants (mentioned in the abstract): "Data were collected from 19 users ..."

This has been added,

I think it would have been interesting/useful adding some information regarding the type of tests used (RT-PCR, RDTs, assays for antibody detection like ELISAs). Considering the results, most of the participants refer to RDTs.

Also, information regarding the types of tests to be used according the number of days after onset of symptoms, and the interpretation of the results according to that (i.e. False negatives)

Thank you, this section of the introduction has been increased.

Regarding case reporting and information transfer (Line 195): Is an epidemiological form/questionnaire used to collect data? Is this done on paper or online? How does communication with the surveillance system occur? how are the communication steps carried out?

Thank you, more of these descriptive results are now provided.

Results
Line 266: It is suggested to use the phrase "low sensitivity tests"
This has been changed

Discussion
It is noteworthy that some physicians do not request laboratory tests because other arboviruses (Zika, chikungunya, etc.) have been reported in Thailand, as well as other pathogens that can cause clinical pictures similar to dengue, highlighting the importance of differential diagnosis. Perhaps this topic could be elaborated in the discussion.

Thank you, an additional section has been added to the discussion.

With respect to the data transfer, it could be added that certain technologies have been used for dengue surveillance. i.e. "Digital Health Interventions in Dengue Surveillance to Detect and Predict Outbreak: A Scoping Review" (DOI: 10.2174/0118749445283264240116070726).

Thank you for highlighting this recent publication, which we have now cited.

Figures

- Figures 1 and 2 show very interesting/relevant data. For clarity, the layouts of these images could be improved, (scheme/diagram and colors) Also, not all components of the background figure are visible and clear.

Thank you we have redrafted these figures.

- Figure 3. Please add the meaning of AFI abbreviation (in the figure caption/footnote)

Added

The "remote-connected serotype-specific diagnostics" box should be connected to some process/box in the figure?

This box is intended to represent the potential solution to information loss and delay. So it sits in the middle and we have left it unconnected. However, we have repositioned and relabelled it, to make it clearer.

You might want to add to this figure that currently the information does not go back (downwards) to the users (physicians, nurses, researchers).

Thank you, this has been added

What type of activities is done for disease control/prevent transmission?

Thank you, a brief description of these activities has been added.

Limitations

It could be added that the findings are limited to the 19 participants interviewed and so future research examining this topic in in a larger number of participants (representative of the different occupational groups) may have a broader scope.

This caveat has been addded

Formatting

For the final version, could references along the manuscript be included in abbreviated form?
i.e: [20], [21], [22], [23], [24] could be cited as 20-24

Some comments were included in the manuscript.

VERSION 2 - REVIEW

Reviewer 2

Name **Rojas, Alejandra**

Affiliation **Instituto de Investigaciones en Ciencias de la Salud, Universidad Nacional de Asunción, Production**

Date **26-Jul-2024**

COI **I declare that there are no competing interests.**

The suggestions proposed in revision 1 were addressed by the authors. Some new comments/suggestions were included in the manuscript attached (Sections: Introduction, Results, Discussion, Table 2, Figure 2).

Item 15 (Review Checklist). Is the standard of written English acceptable for publication? I cannot fully evaluate this topic because I am not a native speaker.

****The reviewer provided a marked copy with additional comments. Please contact the publisher for full details.**

VERSION 2 - AUTHOR RESPONSE
